# Human and molecular genetics shed lights on fatty liver disease and diabetes conundrum

**DOI:** 10.1002/edm2.179

**Published:** 2020-09-04

**Authors:** Federica Tavaglione, Giovanni Targher, Luca Valenti, Stefano Romeo

**Affiliations:** ^1^ Clinical Medicine and Hepatology Unit Department of Internal Medicine and Geriatrics Campus Bio‐Medico University Rome Italy; ^2^ Department of Molecular and Clinical Medicine Sahlgrenska Academy University of Gothenburg Gothenburg Sweden; ^3^ Section of Endocrinology, Diabetes and Metabolism Department of Medicine University and Azienda Ospedaliera Universitaria Integrata of Verona Verona Italy; ^4^ Department of Pathophysiology and Transplantation Università degli Studi di Milano Milano Italy; ^5^ Translational Medicine Department of Transfusion Medicine and Hematology Fondazione IRCCS Ca’ Granda Ospedale Maggiore Policlinico Milano Italy; ^6^ Clinical Nutrition Unit Department of Medical and Surgical Science Magna Graecia University Catanzaro Italy; ^7^ Department of Cardiology Sahlgrenska University Hospital Gothenburg Sweden

**Keywords:** diabetes, fatty liver disease, human genetics

## Abstract

The causal role of abdominal overweight/obesity, insulin resistance and type 2 diabetes (T2D) on the risk of fatty liver disease (FLD) has robustly been proven. A consensus of experts has recently proposed the novel definition of ‘metabolic dysfunction‐associated fatty liver disease, MAFLD’ instead of ‘nonalcoholic fatty liver disease, NAFLD’, emphasizing the central role of dysmetabolism in the disease pathogenesis. Conversely, a direct and independent contribution of FLD *per se* on risk of developing T2D is still a controversial topic. When dealing with FLD as a potential risk factor for T2D, it is straightforward to think of hepatic insulin resistance as the most relevant underlying mechanism. Emerging evidence supports genetic determinants of FLD (eg *PNPLA3, TM6SF2, MBOAT7, GCKR, HSD17B13*) as determinants of insulin resistance and T2D. However, recent studies highlighted that the key molecular mechanism of dysmetabolism is not fat accumulation *per se* but the degree of hepatic fibrosis (excess liver fat content—lipotoxicity), leading to reduced insulin clearance, insulin resistance and T2D. A consequence of these findings is that drugs that will ameliorate liver fat accumulation and fibrosis in principle may also exert a beneficial effect on insulin resistance and risk of T2D in individuals with FLD. Finally, initial findings show that these genetic factors might be directly implicated in modulating pancreatic beta‐cell function, although future studies are needed to fully understand this relationship.

## Graphical abstract

Emerging evidence supports genetic determinants of fatty liver disease as determinants of insulin resistance and type 2 diabetes. The key molecular mechanism of dysmetabolism is not fat accumulation *per se* but the degree of hepatic fibrosis (excess liver fat content—lipotoxicity).

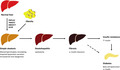

## 1 INTRODUCTION

Fatty liver disease (FLD) is defined by excessive hepatic fat accumulation mainly due to metabolic derangement and excess in alcohol intake.^1^ Abdominal overweight/obesity, insulin resistance and type 2 diabetes (T2D) are among the strongest acquired risk factors for the development of FLD and its progression to advanced fibrosis, cirrhosis and hepatocellular carcinoma.^2^, ^3^, ^4^ The causal role of abdominal overweight/obesity, insulin resistance and T2D on risk of FLD development and progression has robustly been proven.^5^ The opposite, namely a direct and independent contribution of FLD *per se* on risk of developing T2D, is still a controversial topic.

However, it is becoming clear that the link between FLD and T2D is more complex than previously thought. Human genetic variations primarily increasing liver fat content do not have a direct effect on insulin resistance.^6^ Indeed, recent evidence suggests that quality of fat, rather than quantity, is more important in causing the increase in insulin resistance.^6^, ^7^ Furthermore, the effect of gender in the development of FLD should not be dismissed.^8^ A growing body of evidence suggests that gender and its related biological components represent strong determinants of FLD development and progression.^9^ In agreement, also derangement in glucose metabolism has a sexual dysmorphism.^10^, ^11^, ^12^, ^13^, ^14^, ^15^, ^16^ Among the unknown questions, there is also if genetic determinants of FLD interact specifically with sex. Increasing clinical evidence now suggests that FLD may precede and/or promote the development of T2D and other cardiometabolic diseases.^17^ Thus, FLD appears to be a good biomarker for predicting risk of incident T2D, irrespective of established risk factors and may be also used to stratify the risk of cardiometabolic diseases and personalize prevention. When dealing with FLD as a new risk factor for T2D, it is straightforward to think of liver fat content contributing directly to hepatic insulin resistance and diabetes as the most likely mechanism.^18^ However, as will be discussed in greater detail, emerging data are now challenging this notion.

Very recently, a consensus of experts has proposed to replace the ‘nonalcoholic fatty liver disease, NAFLD’ with a more appropriate term, namely ‘metabolic dysfunction‐associated fatty liver disease, MAFLD’.^19^, ^20^ This novel term emphasizes that derangement in hepatic lipid and glucose handling, namely metabolic dysfunction, is the key player in the pathogenesis of chronic liver disease. In particular, they propose a set of novel affirmative criteria for diagnosing MAFLD (mainly based on the presence of overweight/obesity, T2D or other metabolic syndrome traits), irrespective of other concomitant liver diseases. However, this term has not been unanimously accepted^21^ and therefore, in this review we will use the term FLD.

In this review article, we will focus on the contribution of human genetics to the multifaceted and bidirectional relationship between FLD and T2D, highlighting the potential clinical use of FLD for a better risk stratification of T2D and its related chronic vascular complications (mainly cardiovascular and chronic kidney disease).

## 2 EPIDEMIOLOGY

### 2.1 FLD and increased risk of diabetes: epidemiological evidence

A body of evidence shows that FLD, as detected by imaging methods, is an early predictor for the development of incident T2D.^3^, ^4^ In Table 1, we included the observational studies, published in the last 5 years, investigating the association between FLD and risk of incident T2D.^22^, ^23^, ^24^, ^25^, ^26^, ^27^, ^28^, ^29^, ^30^, ^31^, ^32^, ^33^, ^34^, ^35^, ^36^, ^37^, ^38^, ^39^, ^40^ Collectively, all these studies have consistently documented that FLD was strongly associated with an increased risk of incident T2D, independently of age, sex, adiposity measures and other potential confounding factors (Table 1). The increased risk of incident T2D ranged approximately from a 50%^30^ to 3.5‐fold increase^36^ in individuals with FLD, becoming even higher in sex‐stratified analyses.^35^ The significant association between FLD and increased risk of incident T2D was also confirmed among FLD individuals with prediabetes.^39^

**Table 1:** Observational studies published from 2016 to 2020 that assessed the association between FLD (as detected by imaging or biopsy) and the risk of incident type 2 diabetes

Author, year	Study characteristics; follow‐up length	Diagnosis of FLD	Diagnosis of diabetes	Covariate adjustment	Main findings
Chen, 2016^22^	Prospective cohort study of 6,542 (3.2% with FLD) Chinese nondiabetic subjects without known chronic liver diseases; 6 years	Ultrasound	Fasting glucose ≥ 7.0 mmol/L, HbA1c ≥ 6.5% or drug treatment	Age, obesity, hypertriglyceridaemia, IFG	FLD was independently associated with incident diabetes (adjusted HR 2.17, 95% CI 1.6‐3.0)
Li, 2017^23^	Prospective cohort study of 18,111 (31.9% with FLD) Chinese nondiabetic subjects without known chronic liver diseases; 4.6 years	Ultrasound	Fasting glucose ≥ 7.0 mmol/L, clinical history or drug treatment	Age, sex, BMI, waist circumference, alcohol intake, smoking, exercise, family history of diabetes, fasting glucose, triglycerides, total cholesterol	The adjusted HRs for incident diabetes compared with those without FLD were as follows: 1.88 (95% CI 1.6‐2.2) in the mild FLD group and 2.34 (95% CI 1.9‐3.0) in the moderate‐severe FLD group (*P‐trend* < 0.001)
Ma, 2017^24^	Prospective cohort study of 1,051 (17.8% with FLD) United States nondiabetic subjects without known chronic liver diseases; 6.2 years	Computed tomography	Fasting glucose ≥ 7.0 mmol/L or drug treatment	Age, sex, BMI, smoking, exercise, alcohol intake, fasting glucose, changes in BMI and liver phantom ratio during follow‐up	FLD was independently associated with incident diabetes (adjusted OR 2.85, 95% CI 1.4‐6.0, *P* = .006)
Chen, 2017^25^	Prospective cohort study of 132,377 (32% with FLD, 18.1% with chronic liver diseases) Taiwanese nondiabetic subjects; 5.8 years	Ultrasound	Fasting glucose ≥ 7.0 mmol/L, clinical history or drug treatment	Age, obesity, hypertension, dyslipidemia, family history of diabetes, smoking, alcohol intake, exercise, AST, ALT, GGT, ALP	FLD was independently associated with incident diabetes (adjusted HR 2.08, 95% CI 1.9‐2.2, *P* < .001 in men and adjusted HR 2.65, 95% CI 2.4‐2.9, *P* < .001 in women). Exclusion of chronic liver diseases did not attenuate the association
Liu, 2017^26^	Retrospective cohort study of 18,507 (18.8% with FLD) Chinese elderly nondiabetic males without known chronic liver diseases; 5 years	Ultrasound	Fasting glucose ≥ 7.0 mmol/L, 2‐h plasma glucose ≥ 11.1 mmol/L during 75‐g OGTT, clinical history or drug treatment	Age, BMI, ALT, smoking, marriage status, alcohol intake, hypertension, dyslipidemia	FLD was independently associated with incident diabetes (adjusted RR 1.67, 95% CI 1.4‐2.1, *P* < .001)
Björkström, 2017^27^	Retrospective cohort study of 396 (100% with FLD) Swedish nondiabetic subjects without known chronic liver diseases; 18.4 years	Biopsy	Clinical history or drug treatment	Age, sex, BMI, triglycerides	Liver fat content was independently associated with incident diabetes in the fibrosis stages 0‐2 (adjusted HR 1.36, 95% CI 1.0‐1.8; *P* = .03), not in the fibrosis stages 3‐4 (adjusted HR 1.24, 95% CI 0.4‐3.7, *P* = .71)
Tokita, 2017^28^	Retrospective cohort study of 2,408 (11.2% with FLD) Japanese nondiabetic subjects without known chronic liver diseases; 10 years	Ultrasound	Fasting glucose ≥ 7.0 mmol/L, HbA1c ≥ 6.5% or drug treatment	Age, sex, HbA1c, HDL, triglycerides, systolic blood pressure	FLD was independently associated with incident diabetes (*P* = .0001)
Mitsuhashi, 2017^29^	Retrospective cohort study of 17,810 (21.6% with FLD) Japanese nondiabetic subjects without known chronic liver diseases; 5.1 years	Ultrasound	Fasting glucose ≥ 7.0 mmol/L, HbA1c ≥ 6.5%, clinical history or drug treatment	Age, BMI, smoking, exercise, alcohol intake, family history of diabetes, log ALT, fasting glucose	The adjusted HR for incident diabetes compared with those without FLD and MetS were as follows: 2.35 (95% CI 1.9‐2.9, *P* < .001) in the non‐MetS with FLD group, 1.70 (95% CI 1.3‐2.2, *P* < .001) in the MetS without FLD group, and 2.33 (95% CI 1.9‐2.9, *P* < .001) in the MetS with FLD group
Bae, 2018^30^	Retrospective cohort study of 7,849 (46.7% with FLD) Korean nondiabetic subjects without known chronic liver diseases; 4 years	Ultrasound	Fasting glucose ≥ 7.0 mmol/L, HbA1c ≥ 6.5%, clinical history or drug treatment	Age, sex, fasting glucose, HbA1c, BMI, LDL, HDL, triglycerides, systolic blood pressure, HOMA‐IR, smoking	Changes in FLD status were independently associated with incident diabetes. The adjusted HRs compared with those without FLD were as follows: 1.50 (95% CI 1.1‐2.0) in the persistent FLD group and 0.99 (95% CI 0.8‐1.3) in the resolved FLD group
Seko, 2018^31^	Retrospective cohort study of 89 (100% with FLD) Japanese nondiabetic subjects (58% with IGT) without known chronic liver diseases; 5.2 years	Biopsy	Fasting glucose ≥ 7.0 mmol/L, 2‐h plasma glucose ≥ 11.1 mmol/L during 75‐g OGTT, HbA1c ≥ 6.5% or drug treatment	Age, sex, BMI, ferritin, fibrosis stage, NAS, AST/ALT ratio, fasting glucose, 30‐min and 1‐h postload plasma glucose, HbA1c, 1‐h postload insulin, HOMA‐B, HOMA‐IR	Insulin resistance was independently associated with incident diabetes (adjusted HR 40.1, 95% CI 1.4‐119.3, *P* = .033)
Kim, 2018^32^	Retrospective cohort study of 2,920 (31.6% with FLD, 3.5% with diabetes) Korean subjects without known chronic liver diseases; 5.1 years	Ultrasound	Fasting glucose ≥ 7.0 mmol/L, HbA1c ≥ 6.5% or drug treatment	Age, sex, waist circumference, triglycerides, HDL, LDL, uric acid, smoking	FLD was independently associated with incident diabetes. The adjusted HRs compared with the nonobese without FLD group were as follows: 2.69 (95% CI, 1.7‐4.2, *P* < .001) in the nonobese with FLD group, 1.92 (95% CI, 1.1‐3.4, *P* = .022) in the obese without FLD group, and 2.89 (95% CI, 1.7‐4.8, *P* < .001) in the obese with FLD group
Shen, 2018^33^	Prospective cohort study of 41,650 (28.4% with FLD) Chinese nondiabetic subjects without known chronic liver diseases; 3.6 years	Ultrasound	Fasting glucose ≥ 7.0 mmol/L or drug treatment	Age, sex, smoking, exercise, education, family incomes, family history of diabetes, waist circumference, ALT, LDL, HDL, triglycerides, fasting glucose, uric acid, C‐reactive protein, hypertension, metabolic syndrome	The severity of FLD was associated with higher risk of incident diabetes. The adjusted HRs compared with those without FLD were as follows: 1.62 (95% CI 1.5‐1.8) in the whole FLD group, 1.46 (95% CI 1.3‐1.6) in the mild FLD group, 1.92 (95% CI 1.7‐2.2) in the moderate FLD group and 2.66 (95% CI 2.2‐3.3) in the severe FLD group (*P‐trend* < 0.001). Similar associations were observed between FLD and incident IFG
Brunner, 2019^34^	Retrospective cohort study of 808 (14% with FLD, 2.5% with diabetes) United States subjects; 6.2 years	Computed tomography	Fasting glucose ≥ 7.0 mmol/L or drug treatment	Age, sex, BMI, smoking, alcohol intake, liver phantom ratio, fasting glucose, changes in BMI during follow‐up	Increasing liver fat content during follow‐up was independently associated with incident diabetes (adjusted OR 1.68, 95% CI 1.2‐2.3, *P* = .001)
Okamura, 2019^35^	Retrospective cohort study of 15,464 (17.7% with FLD) Japanese nondiabetic subjects without known chronic liver diseases; 5.1 years	Ultrasound	Fasting glucose ≥ 7.0 mmol/L, HbA1c ≥ 6.5% or clinical history	Age, alcohol intake, smoking, exercise, fasting glucose	FLD was independently associated with incident diabetes (adjusted HR 4.74, 95% CI 1.9‐11.7, *P* = .006 in men and adjusted HR 14.0, 95% CI 7.2‐27.1, *P* < .001 in women). The clustering of obesity, visceral obesity and FLD markedly increased the risk of developing diabetes (adjusted HR 10.5, 95% CI 8.0‐13.8, *P* < .001 in men and adjusted HR 30.0, 95% CI 18.0‐50.0, *P* < .001 in women)
Cho, 2019^36^	Retrospective cohort study of 2,726 (30.3% with FLD) Korean nondiabetic subjects without known chronic liver diseases; 62.2 months	Ultrasound	Fasting glucose ≥ 7.0 mmol/L, HbA1c ≥ 6.5% or drug treatment	Age, sex, BMI, fasting glucose, ALT	Changes in FLD status were independently associated with incident diabetes. The adjusted HRs compared with those without FLD were as follows: HRs 3.59 (95% CI 2.1‐6.3, *P* < .001) in the persistent FLD group, 1.94 (95% CI 1.1‐3.5, *P* = .026) in the incident FLD group and 1.21, 95% CI, 0.4‐3.6, *P* = .733) in the resolved FLD group
Sung, 2019^37^	Retrospective cohort study of 70,303 (13.1% with FLD) Korean nondiabetic subjects without known chronic liver diseases; 3.7 years	Ultrasound	Fasting glucose ≥ 7.0 mmol/L, HbA1c ≥ 6.5%, clinical history or drug treatment	Age, education, exercise, smoking, alcohol intake, centre, year, family history of diabetes, waist circumference, BMI, triglycerides, LDL, drugs for hypertension and hyperlipidaemia	FLD was independently associated with incident diabetes (adjusted HR 2.17, 95% CI 1.6‐3.0 in men and adjusted HR 2.86, 95% CI 1.5‐5.5 for women)
Wang, 2019^38^	Retrospective cohort study of 10,064 (5.4% with FLD) Japanese nondiabetic subjects without known chronic liver diseases; 6 years	Ultrasound	Fasting glucose ≥ 7.0 mmol/L, HbA1c ≥ 6.5% or clinical history	Age, sex, BMI, alcohol intake, smoking, HbA1c	FLD was independently associated with incident diabetes (adjusted HR 2.52, 95% CI 1.6‐4.0, *P* < .001)
Lee, 2019^39^	Retrospective cohort study of 6,240 (45.4% with FLD) Korean prediabetic subjects without known chronic liver disease; 4.3 years	Ultrasound	Fasting glucose ≥ 7.0 mmol/L, HbA1c ≥ 6.5% or drug treatment	Age, sex, BMI, smoking, alcohol intake, ALT, triglycerides, HDL, systolic blood pressure, HbA1c	FLD was independently associated with incident diabetes (adjusted RR 1.81, 95% CI, 1.5‐2.2, *P* < .001)
Nasr, 2020^40^	Prospective cohort study of 106 (100% with FLD) Swedish nondiabetic subjects without known chronic liver diseases; 23.2 years	Biopsy	Fasting glucose ≥ 7.0 mmol/L with drug treatment or 2‐h plasma glucose ≥ 11.1 mmol/L during 75‐g OGTT	Age, BMI, histologic fibrosis stage	Liver fat content was independently associated with incident diabetes (adjusted HR 1.03 per 1% increase, 95% CI 1.0‐1.1, *P* = .02)

Abbreviations: ALP, alkaline phosphatase; ALT, alanine aminotransferase; AST, aspartate aminotransferase; BMI, body mass index; CI, confidence interval; FLD, fatty liver disease; GGT, gamma‐glutamyl transferase; HbA1c, haemoglobin A1c; HDL, high‐density lipoprotein; HOMA‐B, homeostasis model assessment of beta‐cell function; HOMA‐IR, homeostasis model assessment of insulin resistance; HR, hazard ratio; IFG, impaired fasting glycaemia; IGT, impaired glucose tolerance; LDL, low‐density lipoprotein; MetS, metabolic syndrome; NAS, NAFLD activity score; OGTT, 75‐g oral glucose tolerance test; T2D, type 2 diabetes.

Notably, the increase in the risk of incident T2D was found to be proportional to the severity of liver steatosis assessed by ultrasonography or computed tomography.^23^, ^33^, ^34^ For example, in a large prospective cohort study of 18,111 Chinese nondiabetic subjects, Li *et al* showed that the incidence rates of T2D at 4.6‐year follow‐up progressively increased with the ultrasonographic severity of FLD at baseline, accounting for 18.1% of incident T2D cases in the moderate‐severe FLD group, 10.6% in the mild FLD group and 4.7% in the normal group, respectively (*P* < .001). In the multivariable Cox regression analysis, the adjusted hazard ratios (HRs) for incident T2D were, respectively, 2.34 (95% CI 1.9‐3.0) and 1.88 (95% CI 1.6‐2.2) in individuals belonging to the moderate‐severe and mild FLD groups, when compared with those in the non‐FLD group (*P‐trend* < 0.001).^23^

Similarly, in a prospective cohort study of 41,650 Chinese nondiabetic individuals followed for a mean period of 3.6 years, it has been reported that FLD on ultrasonography was independently associated with increased incidence of both T2D (adjusted HR 1.62, 95% CI 1.5‐1.8) and prediabetes (adjusted HR 1.12, 95% CI 1.1‐1.2). In particular, compared with subjects without FLD, the HRs for T2D development were significantly greater in those belonging to the severe FLD group (adjusted HR 2.66, 95% CI 2.2‐3.3), the moderate (adjusted HR 1.92, 95% CI 1.7‐2.2) or mild (adjusted HR 1.46, 95% CI 1.3‐1.6) FLD groups.^33^

Interestingly, in a retrospective cohort study of 2,726 South Korean nondiabetic individuals, Cho *et al* have assessed the risk of incident T2D during 62 months of follow‐up in the following three subgroups of subjects: (1) those with persistent FLD on ultrasonography both at baseline and at follow‐up; (2) those with newly diagnosed FLD at follow‐up; and (3) those with FLD resolution at follow‐up examination. Notably, these authors found that compared with individuals without FLD, the risk of incident T2D was remarkably greater in those with persistent FLD (adjusted HR 3.59, 95% CI 2.1‐6.3, *P* < .001) and those who developed incident FLD (adjusted HR 1.94, 95% CI 1.1‐3.5, *P* = .026) over the follow‐up period. Conversely, the risk of incident T2D was not increased in those who resolved FLD at follow‐up (adjusted HR 1.21, 95% CI, 0.4‐3.6, *P* = .733).^36^

Similarly, in a retrospective cohort study of 7,849 South Korean nondiabetic individuals who were followed for a mean period of 4 years, Bae *et al* reported that the persistence of FLD on ultrasonography was independently associated with an approximately 50% increased risk of incident T2D, whereas the risk of individuals who resolved FLD over the follow‐up was essentially superimposable to that of individuals without FLD.^30^

Notably, Mitsuhashi *et al* have also shown that FLD was a stronger risk factor for incident T2D than the presence of metabolic syndrome (MetS) without fatty liver. Indeed, in a population‐based cohort study of over 17,000 Japanese nondiabetic individuals enrolled in a healthy check‐up programme for more than 5 years, the authors found that the incidence rates of T2D were 1.7% in non‐MetS individuals without FLD, 8.3% in individuals with FLD alone, 12.5% in those with MetS alone and 21.2% in those with both conditions, respectively. Compared with the normal group, the adjusted HRs for incident T2D were 2.35 (95% CI 1.9‐2.9) in non‐MetS individuals with FLD, 1.70 (95% CI 1.3‐2.2) in those with MetS alone and 2.33 (95% CI 1.9‐2.9) in those with both MetS and FLD, respectively. Additionally, patients with FLD (irrespective of coexistence of MetS) had a ~ 38% increased risk of developing T2D compared to those with MetS alone.^29^

Using the same population‐based cohort, Okamura *et al* have subsequently shown that FLD *per se* had the strongest adverse effect on risk of incident T2D (adjusted HR 4.74, 95% CI 1.9‐11.7, in men and adjusted HR 14.0, 95% CI 7.2‐27.1, in women) compared with either obesity without FLD (adjusted HR 1.85, 95% CI 1.1‐3.3, in men and adjusted HR 1.79, 95% CI 0.2‐13.2, in women) or visceral obesity without FLD (adjusted HR 3.41, 95% CI 2.5‐4.6, in men and adjusted HR 2.30, 95% CI 0.9‐6.1, in women). As expected, the clustering of these three conditions (obesity, visceral obesity and FLD) markedly increased the risk of incident T2D (adjusted HR 10.5, 95% CI 8.0‐13.8, in men and adjusted HR 30.0, 95% CI 18.0‐50.0, in women).^35^

In a retrospective cohort study of 396 Swedish nondiabetic adults with biopsy‐confirmed FLD, Björkström *et al* have reported that the incidence rate of T2D was significantly higher among subjects with fibrosis stages 3‐4 than among those with fibrosis stages 0‐2 (51% vs. 31%) over a mean follow‐up of 18.4 years.^27^ Subsequently, in a cohort study of 106 Swedish nondiabetic subjects with biopsy‐proven FLD followed for over 20 years, Nasr *et al* from the same research group have observed that the severity of hepatic steatosis, quantitatively measured by stereological point counting, was independently associated with increased T2D incidence (adjusted HR 1.03 per 1% increase, 95% CI 1.0‐1.1).^40^

In a small retrospective cohort study of 89 Japanese nondiabetic subjects (58% with IGT) with biopsy‐confirmed FLD, Seko *et al* have shown that HOMA‐estimated insulin resistance was the strongest independent predictor of incident T2D over a 5.2‐year follow‐up (adjusted HR 40.1, 95% CI 1.4‐119.3).^31^ Noteworthy, a recent combined meta‐analysis and bias analysis including more than 240,000 middle‐aged individuals (mostly of Asian ethnicity) has provided further strong evidence for a causal relationship between FLD and risk of T2D.^41^

Collectively, all these epidemiological studies support the notion that FLD (defined radiologically or histologically) is strongly associated with an increased risk of incident T2D in different ethnic populations and that the magnitude of risk of incident T2D parallels the underlying severity of FLD. However, there are some important limitations to be considered. First, most of the aforementioned observational studies have a retrospective design and are heterogeneous in terms of demographic characteristics, length of follow‐up, covariates included in multivariable regression analyses, as well as severity of FLD. Second, most of the studies included individuals from Asian countries (especially China and South Korea). Third, only few of these studies (Björkström *et al*,^27^ Seko *et al* ^31^ and Nasr *et al* ^40^) have used liver biopsy for diagnosing and staging FLD. Finally, the large majority of studies—except for Liu *et al*,^26^ Seko *et al* ^31^ and Nasr *et al* ^40^—did not perform 75‐g oral glucose tolerance test for the diagnosis of diabetes.

Additional larger prospective cohort studies performed on different ethnic groups, considering also the genetic determinants for FLD, are certainly needed to better define the magnitude of risk of incident T2D associated with FLD.

### 2.2 FLD and risk of T2D chronic complications: epidemiological evidence

The global prevalence of FLD diagnosed by ultrasonography and magnetic resonance spectroscopy among individuals with T2D is currently estimated to be approximately 55%, with the highest rates reported from Europe (68%) and West Asia (67%), followed by South Asia (58%), Latin America (57%), East Asia (52%), the United States (52%) and Africa (30%).^42^ These rates for the global FLD prevalence are nearly twice those observed in the general population from the same regions.^42^, ^43^ Similarly, the global prevalence of histologically proven nonalcoholic steatohepatitis (NASH) and advanced fibrosis among individuals with FLD and T2D is very high, accounting for 37% and 17%, respectively.^42^

Additionally, T2D has been adversely related to the onset of FLD long‐term complications, such as cirrhosis, hepatocellular carcinoma, liver‐related mortality and all‐cause mortality.^44^, ^45^, ^46^, ^47^, ^48^ In this context, T2D seems to be not only a major driven of FLD global burden but also an important risk factor for liver disease progression.

A detailed discussion of the link between FLD and risk of chronic vascular complications of diabetes is beyond the scope of this review article. In brief, the coexistence of FLD and T2D increases not only the risk of developing the more severe forms of FLD (advanced fibrosis, cirrhosis and hepatocellular carcinoma), but also the risk of developing chronic vascular complications of diabetes. Indeed, to date, a number of large population‐based and hospital‐based cohort studies reported an increased incidence of fatal and nonfatal cardiovascular events in individuals with FLD, across a wide range of disease spectra, including T2D.^49^, ^50^ For instance, a prospective nested case‐control study in 744 T2D outpatient individuals without known cardiovascular and or chronic liver damage at baseline demonstrated that those with ultrasound‐detected FLD had a nearly two‐fold increased risk of major adverse cardiovascular events over a follow‐up period of 5 years. Notably, this association was independent of traditional cardiovascular risk factors, diabetes‐related variables and use of hypoglycaemic, antihypertensive, lipid‐lowering and antiplatelet medications.^51^ Similar results were also confirmed in a subsequent larger cohort study of 2,103 outpatients with T2D with a longer follow‐up period (6.5 years).^52^ Accumulating evidence also suggests that FLD is associated with valvular heart disease (mainly aortic‐valve sclerosis) and increased risk of cardiac arrhythmias (mainly permanent atrial fibrillation), especially in individuals with T2D.^53^, ^54^ This supports the notion that the diagnosis of FLD identifies a subset of subjects at higher risk of cardiovascular disease over time.^55^

In the last decade, a growing body of epidemiological evidence also suggests that FLD is significantly associated with an increased prevalence and incidence of microvascular complications of diabetes, especially with chronic kidney disease.^56^ For instance, in the Valpolicella Heart Diabetes Study cohort involving 1,760 T2D outpatients with normal kidney function at baseline, the presence of ultrasound‐diagnosed FLD was associated with an increased risk of incident chronic kidney disease (CKD stage ≥ 3) over a follow‐up period of 6.5 years, independently of established renal risk factors, diabetes duration, glycaemic control and use of medications.^57^ A recent updated meta‐analysis of nine observational studies (including a total of nearly 96,500 adult individuals) confirmed that FLD is associated with a nearly 40% increase in the long‐term risk of incident CKD stage ≥ 3 (ie defined as occurrence of estimated glomerular filtration rate < 60 ml/min/1.73m^2^, with or without accompanying overt proteinuria). In subgroup analyses, the significant association between FLD and increased risk of CKD was particularly evident among patients with T2D and FLD.^58^

However, despite the growing epidemiological evidence that links FLD with the long‐term risk of chronic vascular complications of diabetes, a causal relationship between these two diseases remains to be demonstrated. Additional larger prospective studies in different ethnic populations and translational studies are needed to firmly establish whether FLD (especially in its more advanced forms) actively contributes to the increased risk of macrovascular and microvascular complications observed among patients with T2D and FLD.

## 3 HUMAN GENETICS

### 3.1 Common genetic variants associated with risk of FLD

In the last decade, several common genetic variants have been reported to confer increased genetic susceptibility to or protection against FLD.^59^ Notably, these common genetic variants had a several fold larger effect if compared to common variants of susceptibility in other complex disease traits, including T2D or obesity. A detailed discussion of the association between rare genetic variants of FLD and risk of insulin resistance and diabetes is beyond the scope of this review article. Briefly, rare mutations in *apolipoprotein B (APOB)* predispose to familial hypobetalipoproteinaemia and progressive liver disease due to impaired triglycerides assembly into very low‐density lipoproteins and failure to secrete triglycerides from the liver.^60^ Consistently with common genetic variations, despite higher liver fat content, the risk of insulin resistance and diabetes seems not to be greatly increased in carriers of *APOB* variants.^61^, ^62^, ^63^, ^64^, ^65^ Moreover, although the coexistence of obesity, visceral adiposity and insulin resistance promotes the development of hepatic fat accumulation in these subjects, familial hypobetalipoproteinaemia represents a condition that *per se* leads to higher degree of FLD.^66^, ^67^ In this section, we will discuss the evidence of an association between common genetic variants of FLD and T2D or insulin resistance.

### 3.2 Patatin‐like phospholipase domain‐containing 3

To date, the *patatin‐like phospholipase domain‐containing 3 (PNPLA3)* rs738409 encoding for an isoleucine to methionine substitution at position 148 (I148M) of the protein is the most robust genetic determinant of FLD.^68^, ^69^ This genetic variant is associated with insulin resistance or T2D mainly in individuals with obesity but not in those with normal weight.^68^, ^70^, ^71^, ^72^, ^73^, ^74^, ^75^, ^76^ A possible reason for this association is that obesity uncovers the effect of the *PNPLA3* variant, increasing its effect size.^73^, ^77^ Additionally, quality of intrahepatic lipids, rather than quantity, may exert a major impact on the development of insulin resistance and glucose intolerance.^6^, ^7^, ^78^, ^79^, ^80^, ^81^, ^82^ In particular, in metabolically related FLD, but not in *PNPLA3*‐related FLD, the liver was found to be predominantly enriched with saturated triglycerides and with markers of *de novo* ceramides synthesis.^6^, ^7^ Notably, ceramides have been strongly associated with hepatic insulin resistance, thus supporting their key role in the pathogenesis of metabolically related FLD.^6^, ^7^, ^78^ On the other side, in *PNPLA3*‐associated FLD the quality of triglycerides shifted towards polyunsaturated fatty acids.^7^

However, there are also some studies showing a significant relationship between *PNPLA3* I148M polymorphism and greater insulin resistance in nonobese individuals from Taiwan and South Korea.^83^, ^84^ In addition, in a prospective cohort study of 2,189 Chinese middle‐aged and elderly individuals with a follow‐up of 4.2 years, Xia *et al* showed that the *PNPLA3* rs738409 was significantly associated with lower risk of incident T2D.^85^ Furthermore, in a study of Brazilian individuals with T2D, Machado *et al* reported that the *PNPLA3* I148M variant was significantly correlated to a better glycaemic control.^86^ All these data suggest that in addition to obesity there are also other factors possibly related to ethnicity that can modulate the effect of the *PNPLA3* genetic variant on T2D risk. Further studies are needed to establish the magnitude of genetic and environmental risk factors in FLD pathogenesis and to better characterize the different clinical FLD phenotypes resulting from their interactions.

### 3.3 Transmembrane 6 superfamily member 2

A body of evidence shows that the rs58542926 in *transmembrane 6 superfamily member 2 (TM6SF2)* (E167K) is a robust genetic determinant of FLD,^87^, ^88^, ^89^ inducing a reduction in APOB100 containing lipoprotein lipidation and secretion.^90^, ^91^

Furthermore, studies have also investigated the relationship between FLD, insulin sensitivity and T2D among individuals carrying the *TM6SF2* E167K. As for the *PNPLA3* I148M, lines of evidence have described the *TM6SF2* E167K as a potential risk variant for T2D development,^92^, ^93^ mainly linked to increased hepatic and adipose insulin resistance and impaired pancreatic beta‐cell function.^94^ On the other hand, *TM6SF2* E167K has been reported to be associated with preserved insulin sensitivity, estimated by HOMA‐IR and adipose insulin resistance or measured by hyperinsulinaemic euglycaemic clamp.^84^, ^95^

### 3.4 Membrane bound O‐acyltransferase domain‐containing protein 7

The membrane bound O‐acyltransferase domain‐containing protein 7 (MBOAT7) is a 6‐transmembrane domain protein^96^ that promotes the remodelling of membrane phosphatidylinositol with polyunsaturated fatty acids.^96^, ^97^, ^98^, ^99^ Depletion of MBOAT7 increases liver fat content by inducing hepatic synthesis of triglycerides fueled by an accelerated turnover of phosphatidylinositol.^100^ Hyperinsulinaemia also contributes to liver fat accumulation by enhancing hepatic MBOAT7 down‐regulation, independently of *MBOAT7* rs641738 genotype,^99^ thus suggesting that MBOAT7 activity might be influenced by insulin signalling pathways.

To date, there are very few studies examining the effect of *MBOAT7* rs641738 on T2D‐related metabolic traits. Viitasalo *et al* did not find any association of *MBOAT7* rs641738 with plasma glucose and insulin levels among Caucasian obese children.^101^ Similarly, no association was found between the *MBOAT7* rs641738 and HOMA‐estimated insulin resistance among Asian adult individuals.^84^ However, in a multiethnic cohort of 860 obese youths, Umano *et al* showed that *MBOAT7* rs626283 (ie a genetic variant in strong linkage disequilibrium with the *MBOAT7* rs641738) was associated with both hyperisulinaemia and impaired insulin sensitivity in European individuals, but not in Hispanics and African Americans.^102^

### 3.5 Glucokinase regulator

The rs1260326 in *glucokinase regulator (GCKR)* (P446L) reduces GCKR ability to inhibit glucokinase, resulting in constitutive activation of glucose uptake and increased hepatic *de novo* lipogenesis.^103^ This results in the occurrence of FLD with lower insulin resistance and decreased risk of T2D as shown in several ethnic groups, mostly European and Asian populations.^104^, ^105^, ^106^, ^107^, ^108^, ^109^, ^110^, ^111^, ^112^, ^113^, ^114^, ^115^, ^116^

Notably, as for other genetic variants, a *GCKR*‐related protection against development of T2D was not observed in African American individuals,^113^, ^114^, ^116^ supporting that the impact of *GCKR* variant on T2D risk and its related clinical traits might differ depending on ethnicity. Moreover, the association of the *GCKR* variant with fasting glucose, insulin levels and insulin sensitivity seems to be less pronounced in children or adolescents compared to adults, suggesting that the *GCKR*‐induced hypoglycaemic effect might become more evident with increasing age.^117^, ^118^ Unexpectedly, the rs1260326 or rs780094 (an intronic variant in high linkage disequilibrium) in *GCKR* gene variants have been associated with increased 2‐hour postload plasma glucose levels.^106^, ^114^, ^119^ Finally, inconsistent results have been reported regarding the association between *GCKR* polymorphisms and pancreatic beta‐cell function, as estimated by the HOMA‐B index.^106^, ^110^, ^114^

### 3.6 Hydroxysteroid 17‐beta dehydrogenase 13

The loss‐of‐function rs72613567:TA in *hydroxysteroid 17‐beta dehydrogenase 13 (HSD17B13)* was recently found to protect against the development and progression of both alcoholic and nonalcoholic chronic liver disease, while showing no association with simple steatosis.^120^, ^121^, ^122^ It has been hypothesized that the *HSD17B13* rs72613567:TA may result in defective HSD17B13 enzymatic activity, leading to impaired synthesis of several proinflammatory lipid species (eg leukotriene B4) into the liver.^120^ However, the exact molecular mechanism(s) and the protein function need further investigation.

Similarly, it is still not known whether the *HSD17B13* gene locus influences susceptibility to T2D and insulin resistance. A study by Luukkonen *et al* have recently reported that in European nondiabetic individuals, the *HSD17B13* rs72613567:TA was not significantly associated with changes in fasting glucose and insulin levels or insulin sensitivity, as directly quantified by euglycaemic hyperinsulinaemic clamp technique.^123^

### 3.7 Causal relationships between FLD, insulin resistance and diabetes: Mendelian randomization studies

In the last few years, an increasing number of studies have applied a Mendelian randomization approach to establish a possible causal relationship between FLD and its related metabolic traits, that is insulin resistance and T2D.^89^, ^124^

**Figure 1:**
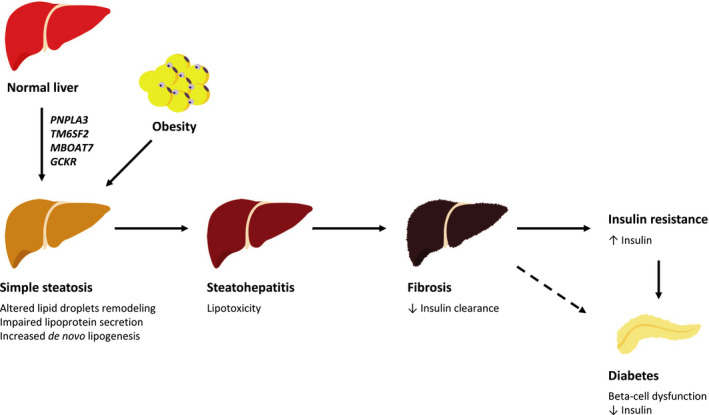
Causal relationship between genetically determined fatty liver disease, insulin resistance and diabetes. A Mendelian randomization study published by Dongiovanni *et al* ^89^ showed that: 1) genetically determined fatty liver disease (FLD) is causally associated with insulin resistance in individuals at risk of progressive liver disease (eg those with suspected NASH or severe obesity); 2) impairment of insulin sensitivity is mediated by increased hepatic fibrosis (excess liver fat content—lipotoxicity). Similarly, a Mendelian randomization study by Liu *et al* ^124^ confirmed that genetically determined FLD causes the development of type 2 diabetes (T2D), although the underlying molecular mechanism(s) has yet to be entirely elucidated. In accord with the well‐recognized link between cirrhosis and increased T2D onset,^126^ the association between genetically determined FLD and enhanced risk of incident T2D might again be largely mediated by increased hepatic fibrosis

Interestingly, it has been shown that the presence of genetically determined fatty liver (by using a genetic risk score including *PNPLA3, TM6SF2, GCKR* and *MBOAT7* variants) was causally associated with greater insulin resistance, as estimated by HOMA‐IR, in individuals at risk of progressive liver disease (ie those with suspected NASH or severe obesity), but not in the general population.^89^ However, it should be noted that as reported by Stender *et al* these genetic variants strongly interact with obesity^125^ and, therefore, it is not surprising that the deleterious metabolic effect of these genetic variants was observed principally among those at higher risk for FLD. Moreover, this study also suggested that FLD *per se* does not directly cause insulin resistance, but the risk is mainly mediated by the degree of liver fibrosis, in other words by the duration and severity of liver disease (Figure 1).^89^

Within this context, hyperinsulinaemia might be secondary to intrahepatic accumulation of specific lipotoxic species in addition to fibrosis‐induced defect in hepatic insulin clearance.^59^ Similarly, given the well‐recognized association between cirrhosis and increased risk of incident T2D,^126^ it would be not surprising if genetically related FLD may cause pancreatic beta‐cell dysfunction through the same underlying mechanism, that is advanced liver fibrosis. However, this issue has yet to be studied in greater detail in future studies. It is worth noting that the accuracy of Mendelian randomization methodology can be compromised by the pleiotropic effects of genetic variants, although this disadvantage is largely minimized by using polygenic risk scores. Another limitation of human‐based studies is partly due to the presence of multiple potential confounding factors (eg comorbidities or use of certain medications) that may weaken or mask the specific genetic associations. For example, the coexistence of severe obesity was found to strongly influence the impact of *PNPLA3* I148M on systemic insulin sensitivity.^70^ Experimental studies conducted in animal models may help to stem these issues. To support this, a recent experimental study published by Liu *et al* reported that *PNPLA3* I148M was associated with chronic hyperglycaemia and increased visceral adiposity, but not with insulin resistance. Interestingly, the authors proposed that *PNPLA3‐*induced reduction in glucose tolerance was largely mediated by pancreatic chronic inflammation, leading to impaired pancreatic insulin and glucagon secretion.^124^ Taken all this together, it would appear that genetically determined liver steatosis does not carry the same diabetogenic risk associated with the metabolically determined liver steatosis. Moreover, quality of intrahepatic lipids, rather than quantity, decides whether the accumulation of fat in the liver will result in changes in glucose metabolism rather than only a deleterious effect for the hepatocyte.

Based on this evidence, it is likely that the use of drugs that will ameliorate liver steatosis and fibrosis in principle should also exert a beneficial effect on insulin resistance and risk of T2D associated with FLD. Currently, several pharmacological therapies have shown promising results in improving liver fat content and inflammation, such as the peroxisome proliferator‐activated receptor γ (PPAR‐γ) agonist pioglitazone and the glucagon‐like peptide 1 (GLP‐1) receptor agonist liraglutide.^127^, ^128^, ^129^, ^130^ In addition to these well‐known antidiabetic drugs, the stearoyl CoA desaturase‐1 (SCD1) modulator aramchol showed improvement in hepatic steatosis and glycaemic control in individuals with prediabetes or T2D and biopsy‐proven NASH (NCT02279524). On the other hand, despite ameliorating hepatic steatosis and fibrosis, the farnesoid X receptor (FXR) agonist obeticholic acid was found to increase a) insulin resistance, estimated by HOMA‐IR and b) circulating levels of low‐density lipoproteins, resulting in a more proatherogenic profile.^131^ Similarly, the chemokine receptor (CCR) 2/5 antagonist cenicriviroc, which showed a primary antifibrotic activity, appears to be likely metabolically neutral.^132^, ^133^ However, larger phase 3 clinical trials are required to further validate these results. Finally, the pleiotropic effects of genetic factors and of drug pathways should be borne in mind when prescribing a drug for individuals with FLD.

### 3.8 Effect of FLD genetics on T2D chronic complications

To date, emerging evidence supports the existence of a significant relationship between some genetic determinants of FLD and susceptibility to diabetic nephropathy, although the topic needs to be further explored.^134^ Notably, the *PNPLA3* I148M has been associated with lower estimated glomerular filtration rate and increased risk of chronic kidney disease among European individuals with T2D.^135^, ^136^ Interestingly, the significant association between the *PNPLA3* I148M variant and increased risk of kidney dysfunction was independent of established renal risk factors and severity of FLD, suggesting that the *PNPLA3* I148M might be directly involved in the pathophysiology of diabetic nephropathy. In line with this hypothesis, PNPLA3 expression was found to be high in the renal cortex, mainly in podocytes.^136^ Conversely, the steatogenic allele in *GCKR* locus seems to protect against the development of chronic kidney disease among T2D individuals,^137^, ^138^ consistently with the *GCKR*‐related hypoglycaemic effect observed in nondiabetic individuals.

Some evidence also suggests that *PNPLA3* and *TM6SF2* gene variants may protect against cardiovascular risk, whereas variants in *GCKR* are associated with increased risk of cardiovascular disease, perhaps mediated by a decrease in the atherogenic dyslipidemia in both *PNPLA3* and *TM6SF2* carriers and an increase in the atherogenic dyslipidemia in *GCKR* carriers.^139^ However, further research is needed to clarify whether ‘genetic‐related FLD’ and ‘metabolic‐related FLD’ exert differential effects on risk of major adverse cardiovascular events.^49^, ^140^

## 4 CONCLUSIONS AND FUTURE PERSPECTIVES

New insights by molecular human genetics robustly support that FLD is causally associated with dysmetabolism and T2D.^89^, ^124^ Recent studies highlighted that the key molecular mechanism of dysmetabolism is not fat accumulation *per se* but the degree of hepatic fibrosis (excess liver fat content—lipotoxicity), leading to reduced insulin clearance, insulin resistance and T2D.^59^ Notably, initial findings show that these genetic factors might be directly implicated in modulating pancreatic beta‐cell function,^124^ although future studies are needed to fully understand this relationship. In this context, it is worth noting that a consensus of experts has recently proposed novel criteria for diagnosing MAFLD (mainly based on the presence of overweight/obesity, T2D or other metabolic syndrome traits), irrespective of other concomitant liver diseases.^19^, ^20^ We believe that this novel definition is the first attempt to define the complexity of FLD and its heterogeneous clinical phenotypes, paving the way for a more fit design of clinical trials that will lead to precision medicine. Finally, it is also reasonable to speculate that the quantitative assessment of liver fat content by novel unconventional methods and the discovery of specific biomarkers of hepatic lipotoxicity will provide a better opportunity to improve the overall risk prediction of incident T2D in all individuals with FLD.

## CONFLICT OF INTEREST

The authors declare no conflict of interest.

## AUTHOR CONTRIBUTIONS

All authors conceived and designed the review, were involved in drafting and revising the manuscript and approved the final version prior to submission.

## Data Availability

Data sharing is not applicable to this article as no new data were created or analysed in this study.
